# Polyploidy Did Not Predate the Evolution of Nodulation in All Legumes

**DOI:** 10.1371/journal.pone.0011630

**Published:** 2010-07-16

**Authors:** Steven B. Cannon, Dan Ilut, Andrew D. Farmer, Sonja L. Maki, Gregory D. May, Susan R. Singer, Jeff J. Doyle

**Affiliations:** 1 United States Department of Agriculture-Agricultural Research Service, Corn Insects and Crop Genomics Research Unit, Iowa State University, Ames, Iowa, United States of America; 2 Department of Plant Biology, Cornell University, Ithaca, New York, United States of America; 3 National Center for Genome Resources, Santa Fe, New Mexico, United States of America; 4 Department of Biology, Carleton College, Northfield, Minnesota, United States of America; Michigan State University, United States of America

## Abstract

**Background:**

Several lines of evidence indicate that polyploidy occurred by around 54 million years ago, early in the history of legume evolution, but it has not been known whether this event was confined to the papilionoid subfamily (Papilionoideae; e.g. beans, medics, lupins) or occurred earlier. Determining the timing of the polyploidy event is important for understanding whether polyploidy might have contributed to rapid diversification and radiation of the legumes near the origin of the family; and whether polyploidy might have provided genetic material that enabled the evolution of a novel organ, the nitrogen-fixing nodule. Although symbioses with nitrogen-fixing partners have evolved in several lineages in the rosid I clade, nodules are widespread only in legume taxa, being nearly universal in the papilionoids and in the mimosoid subfamily (e.g., mimosas, acacias) – which diverged from the papilionoid legumes around 58 million years ago, soon after the origin of the legumes.

**Methodology/Principal Findings:**

Using transcriptome sequence data from *Chamaecrista fasciculata*, a nodulating member of the mimosoid clade, we tested whether this species underwent polyploidy within the timeframe of legume diversification. Analysis of gene family branching orders and synonymous-site divergence data from *C. fasciculata*, *Glycine max* (soybean), *Medicago truncatula*, and *Vitis vinifera* (grape; an outgroup to the rosid taxa) establish that the polyploidy event known from soybean and *Medicago* occurred after the separation of the mimosoid and papilionoid clades, and at or shortly before the Papilionoideae radiation.

**Conclusions:**

The ancestral legume genome was not fundamentally polyploid. Moreover, because there has not been an independent instance of polyploidy in the *Chamaecrista* lineage there is no necessary connection between polyploidy and nodulation in legumes. *Chamaecrista* may serve as a useful model in the legumes that lacks a paleopolyploid history, at least relative to the widely studied papilionoid models.

## Introduction

Polyploidy (or “whole genome duplication” WGD) is a major force shaping plant genomes, a generator of mutation and a source of raw material for evolution. It is thought to be a driver of morphological change [1], has been implicated in the origin of the flowering plants [2], and may have had a role in the differential survival of lineages at the Cretaceous-Tertiary extinction event [3]. The deployment of paralogous genes created by segmental or whole genome duplication is thought to have contributed to the origin and diversification of the angiosperm flower [4]. Thus, is it possible that ancient WGD in legumes also contributed the raw material for the evolution of nodulation – the symbiotic association of plants and soil bacteria that is characteristic of most members of the legume family?

**Figure 1:**
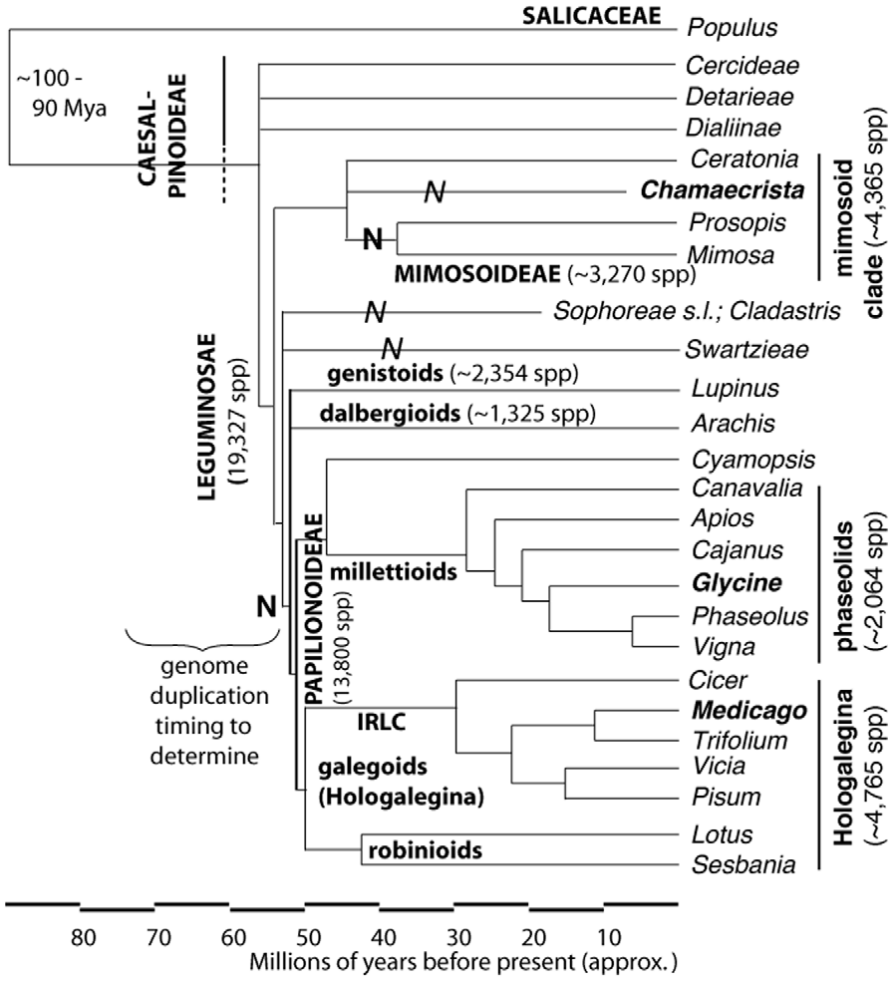
Skeleton phylogeny of the legumes [after 38], [ 39], [ 40], [ 44]. Nodulation in subtended clades is indicated with “N”. Clades with italicized “*N*” contain some nodulating species, but also many non-nodulators. Most sampled genera in the Papilionoideae and Mimosoideae nodulate. The dotted vertical line next to Caesalpinoideae indicates that this subfamily is polyphyletic.

The timing of a polyploidy event early in the radiation of the legumes has been inferred from synonymous-site distances (Ks) in gene sequences [5], [6], and from synteny comparisons within and between genomic sequences [7], [8], [9]. The timing of the early-legume WGD has been narrowed as follows. The WGD predates the separation of the millettioid legumes (including soybean and common bean) from the “cool-season” legumes (in the Hologalegina or “galegoid” clade, including the models *Medicago truncatula* and *Lotus japonicus*) (Figure 1) [5], [8]. On the basis of fossil-calibrated legume phylogenies, the millettioid and galegoid clades are estimated to have separated approximately 54 Mya [10] or 45 Mya [11] (for consistency, the former number and dates for other clades will be used except where noted). Further, *Arachis* (peanut) separated prior to the millettioid and galegoid lineages, around 55 Mya [10]. On the basis of marker data and marker/genomic sequence comparisons, *Arachis* shares the early polyploidy event with the other papilionoids [12]. There are also reports of evidence for the early polyploidy event being present in the genistoids (e.g. *Lupinus*) [13 and Phil McClean, pers. comm] – though timing relative to the genistoid origin is uncertain. Therefore, the early-legume polyploidy predated the origin of at least most of the papilionoids – leaving only a small number of early-diverging papilionoid taxa such as the *Swartzia* and *Cladastris* (and possibly the genistoid) clades uncertain with respect to the early polyploidy event (Figure 1). The next taxonomic landmark with near-complete genomic sequence is *Populus trichocarpa*, in Salicaceae. The Salicaceae are estimated to have separated from the legumes 70–84 Mya [14], [15], [16]. *Populus* does not appear to share the same polyploidy event as legumes [3], [ 8], [but see 17]. This leaves a window of ∼15–30 million years in which the early-legume polyploidy might have occurred: between the origin of the papilionoids, and the common ancestor of the legumes and the Salicaceae.

Determining the relative WGD and speciation timing is important for addressing two hypotheses: that polyploidy might have contributed to rapid diversification and radiation of the legumes near the origin of the family; and that polyploidy might have provided genetic material that led to or contributed to evolution of a novel organ, the nodule. Although symbioses with nitrogen-fixing partners have evolved independently in several lineages in the rosid I clade [18], nodules that host unicellular bacteria (collectively called “rhizobia”) as the symbiont are reported only in the legumes and in the genus *Parasponia* in Ulmaceae [19]. If the mechanisms for symbiotic partnerships were present early during the diversification of the rosid I clade, perhaps through a modification of the infection thread symbioses of the ancient plant-arbuscular mycorrhizal symbiosis [as suggested by 20], then polyploidy early in the legumes may have provided the gene duplicates for further developmental specializations leading to the nodule. An alternative hypothesis is that there were multiple origins of nodulation in the legumes [as suggested by 21].

Determining the WGD timing requires examination of species that may have diverged before the duplication event. Ideally, several species would be chosen along a taxonomic grade, allowing precise timing (Figure 1). If transcript sequences are used to evaluate gene phylogenies, substantial numbers of genes are required in order to overcome the problems of gene loss, non-recovery, and misleading phylogenetic signals. Alternatively, Ks analysis might be used (to track the numbers of accumulated mutations in each lineage); but mutation rates are highly variable among both genes and lineages, so Ks results may not be conclusive, even with large numbers of sampled genes and taxa. Assembling the transcriptomes of numerous species is costly, especially if the goal is the near-full-length transcript sequences required for phylogenetic work. Nevertheless, although having multiple well-sampled species would be ideal, a single well-chosen species should be sufficient at least to determine whether an episode of polyploidy occurred before or after separation of that species from others that are known to have the polyploidy event in their history.

We chose *Chamaecrista fasciculata* because molecular phylogenies place *Chamaecrista* in the large “mimosoid” clade that is sister to the papilionoid clade [22]; the two clades diverged approximately 58 Mya [10]. The clade containing *Chamaecrista* is dominated by mimosoid legumes, the other major group of nodulating legumes outside of the papilionoids [22]. *Chamaecrista* also nodulates, but is not part of the core mimosoid group [11]. Therefore, *Chamaecrista* has a common ancestor with the papilionoids near the origin of the legumes, and a common ancestor with the mimosoids near the origin of the mimosoid clade. Determining whether *Chamaecrista* has the papilionoid WGD therefore will test whether the common ancestor shared with papilionoids was already polyploid, or, alternatively, whether the WGD known from *Glycine* and *Medicago* is confined to papilionoids. Besides being well placed taxonomically to address the question of polyploidy timing early in legume evolution, the species also has characteristics that make it a good candidate as a biological model in several respects in addition to nodulation, including small size, short generation time, self compatibility, and high ecotypic diversity [23].

To determine the relative timings of polyploidy and speciations in the legumes, we sequenced a large portion of the transcriptome of *C. fasciculata* (*Cf*) and examined the phylogenetic placements of a large number of these sequences in gene families relative to sequences from *G. max* (*Gm*), *M. truncatula* (*Mt*), and *V. vinifera* (*Vv*). *Vitis* serves as a useful outgroup because it is in the rosid clade, as an outgroup to both rosid I and rosid II [24], [25], yet has not undergone an additional WGD after its separation from other rosid I taxa [26]. In contrast, poplar, a candidate outgroup in rosid I, has undergone an additional, independent WGD since its separation from the legumes [26], [27], [28]. Comparisons of gene trees from these species enables conclusions about WGD timing in early legume evolution.

## Results

### Strategy

**Figure 2:**
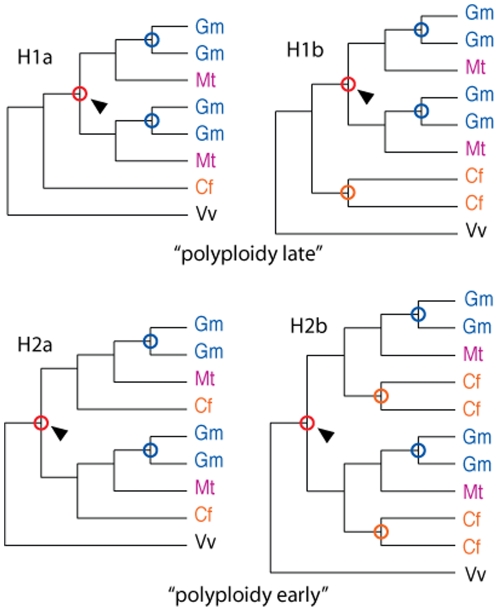
Models of speciation and polyploidy timings. Each figure represents a gene tree that would be expected, assuming no gene losses or additional gains, under the following models: H1  =  polyploidy after the *Chamaecrista* – papilionoid split; H2  =  polyploidy before the *Chamaecrista* – papilionoid split; “a” variants  =  no independent polyploidy in the *Chamaecrista* lineage; “b” variants  =  independent polyploidy in the *Chamaecrista* lineage.

Our primary strategy to distinguish relative dates for WGD and speciation was to examine gene phylogenies from low-copy genes belonging to gene families where there were sufficient representatives from all four species (*Cf*, *Gm*, *Mt*, and *Vv*); and then to score the phylogenies as fitting one of two evolutionary models. The “WGD-late” hypothesis (1) is that WGD occurred within or very shortly before the papilionoids, after the mimosoid (*Cf*)/papilionoid (*Gm*, *Mt*) split; and the “WGD-early” hypothesis (2) is that WGD predated the mimosoid/papilionoid split. It is also possible that the *Cf* lineage has an additional WGD independent of the episode apparent in the papilionoid lineage; this can be tested as a separate hypothesis: “*Cf* WGD”, with two alternatives (a, no WGD vs. b, WGD). The two sets of hypotheses together give four hypotheses, H1a, H1b, H2a, H2b (Figure 2).

A phylogenetic strategy mostly avoids the problems introduced by the imprecision and lineage-specific rates of silent-site changes in Ks analyses. Nevertheless, a phylogenetic approach also presents challenges. Gene families that undergo rapid birth and death or gene conversion may not be suitable for this analysis. Gene families with some rapidly evolving members may not be suitable if these introduce conflicting phylogenetic signals (such as long-branch attraction). Additionally, some families might otherwise be suitable given complete genome (or transcriptome) sequences, but the phylogenies are unusable because of gene loss or data incompleteness. The completeness of the *Cf* transcriptome is not yet known, although one estimate places the proportion of at least partially-sequenced genes at ∼72–86% (see details below). The *Mt* gene set is estimated at 65% complete for the 2007 *Mt*2.0 genome release [29]. Gene sets are estimated to be more than 95% complete for *Gm* [9] and *Vv* [26].

### Sequence characteristics

The diversity of sampled tissues and the sequencing depth gives us confidence that the majority of the *Chamaecrista* transcriptome is captured in our sequence assemblies. The 454 Titanium sequencing generated 367,802 sequences and 124 Mbp total, with 338 bp average read length. The Illumina sequencing generated 132,003,973 passing sequences, each 46 bp long, for a total of 6,069 Mbp. After contigging just the 454 sequences, there were 90,329 sequences and 46,537 Mbp total, with 515 bp average length. Adding the Illumina sequence to the 454 contigs produced fewer, and significantly larger contigs: 54,903 contigs, with average length 599 bp (maximum 5758, minimum 43).

Evaluation of the *Cf* contigs in the context of codon-space alignments suggests that *de novo* assemblies of the large number of 454 and Illumina reads generally gave coherent sequences (e.g., few obvious chimeric or nonsensical sequences were present in the alignments), but also that the contigs contained relatively high numbers of apparently allelic sequences and sequences differing by a few single-base indels. To further collapse these before phylogeny construction, we identified open reading frames, and then re-contigged the resulting sequences. This produced 21,781 sequences, with average length of 463 nt (maximum 3,376, minimum 48). Contig sequences are available in Supplementary File S1.

Several features from the *Cf* sequence statistics are worth highlighting. New sequencing technologies generate large amounts of data: this project produced more than 124 million bases in long reads, and more than 6 billion bases in short reads. The final contigs of 54,903 sequences (average length 599 nt), and 21,781 in-frame, non-redundant contigs (463 nt), can be compared (for point of reference) to the contigs produced from the major *Medicago* Sanger-sequence EST project. The Dana Farber Cancer Institute *Medicago truncatula* EST release 9.0, on 259,642 ESTs, includes 29,273 TCs (“tentative consensus” sequences, or contigs), with average contig length of 1,013 bases [30].

One approximate indication of coverage completeness is the number of *Gm* genes that are matched by *Cf* transcripts, so that *Gm* genes without *Cf* homologs suggest “missing” *Cf* genes. This comparison makes the assumptions that most *Gm* genes have been predicted, that homology can be detected over this evolutionary distance, and that few completely novel genes (i.e. without homology) have arisen in either lineage. The same procedure can be made with *Mt* predicted genes for comparison (from the *Mt*2.0 release, [29]). Using blastx at E-value < =  1e-8, 85.7% and 80.0% of predicted soybean genes have matches to one or more *Cf* contigs or *Mt* genes, respectively; 80.1% and 66.2% have matches to *Cf* contigs or *Mt* genes with percent identity of > = 50%; and 72.7% and 53.7% have matches to *Cf* contigs or *Mt* genes with percent identity of > = 60%. So in this range of parameters, between ∼72 and 86% of *Gm* genes are matched by *Cf* contigs; and over the whole range, *Cf* transcript coverage is greater than for *Mt* gene coverage.

### Clustering and alignment results

We attempted to avoid systematic biases in generating and scoring phylogenetic trees. Sequences were compared for similarity, then clustered, aligned, and used for tree construction. At each stage, we applied filtering criteria selected to retain informative gene families and trees. The initial clustering step produced 11,820 clusters. Clusters were filtered to require at least enough representatives from each species (*Cf* > = 1, *Gm* > = 2, *Mt* > = 1, *Vv* > = 1) to be potentially informative regarding the duplication/polyploidy timing hypotheses. Additionally, we considered only families with no more than 100 sequences. These species-composition filters left 3,330 clusters. After aligning sequences for each cluster and trimming to regions present in at least half of the *Cf* sequences in the alignment, 2,667 alignments remained. Finally, we retained only those alignments that could be put consistently in-frame (with fewer than 0.1% internal stops), and that had an alignment length of at least 60 aa. This left 1,249 final alignments and trees, with 9,861 sequences. The average alignment length was 552.8 nt. All of these sequences were usable for determining basic statistics such as species composition (details below), but only 825 alignments and trees were scorable manually (with 3 or fewer sequence losses) for the four phylogenetic models (Figure 2 and Table S3). These 825 trees resulted in 852 scorable clades (results below), and 180 scorable clades with our automated procedure with 3 or fewer sequence losses. Trimmed alignments and trees are available in Supplementary File S2.

### Transcriptome completeness, gene loss, and consequences

In the 1,249 trees used for mining of phylogenetic patterns, the numbers of sequences from *Cf*, *Mt*, and *Vv* (Table 1) are all within 20% of one another. The counts may be affected by losses in *Mt* (due in some part to the estimated 65% transcriptome completeness in the *Mt* 2.0 assembly version) and *Cf* (with likely non-recovery of some transcripts). Despite these sources of noise in the counts, the effect of recent polyploidy in *Gm* is evident, with the *Gm* count being 2.35 times larger than *Mt*.

**Table 1:** Summary of clade-pattern counts from 1249 informative phylogenetic trees.

A.	Single-sequence counts					
	C	1637				
	M	1877				
	G	4431				
	V	1916				
B.	Pair counts					
	(C,C)	49				
	(M,M)	190				
	(G,G)	1749				
	(V,V)	68				
C.	Automated counting					
		gene loss vs. model			sum,	sum,
Model	0	1	2	3	0–1	0–3
1a	11	62	36	22	73	131
1b	0	0	3	0	0	3
2a	3	9	30	4	12	46
2b	0	0	0	0	0	0
1ab/2ab	3.7	6.9	1.3	5.5	6.1	2.9
D.	Manual counting					
		gene loss vs. model			sum,	sum,
Model	0	1	2	3	0–1	0–3
1a	31	128	116	492	159	**767**
1b	2	4	3	5	6	**14**
2a	2	14	34	19	16	**69**
2b	0	0	0	1	0	**1**
1ab/2ab	16.5	9.4	3.4	24.9	10.3	**11.1**

Columns indicate numbers of missing sequences relative to full patterns for each model. Columns 1 and 2 correspond to counts shown in Table 1; individual counts for the remaining patterns are shown in Tables S2 and S3.

A. Single-sequence counts. The 1,249 scored trees contained these numbers of *Chamaecrista*, *Medicago*, *Glycine*, and *Vitis* sequences.

B. Pair counts. The scored trees contained these numbers of simple sequence pairs. The elevated number of (G,G) is consistent with recent genome duplication in *Glycine*.

C. Automated counting, using pattern matching; numbers in cells are the number of trees or, for more complex trees, clades scored as having either strong or weak support for a given model (hypothesis). Columns: numbers of gene losses for scored trees (up to 3) relative to the indicated models. Rows: models.

D. Manual counting, using visual inspection of all trees. Columns and rows are the same as in C. Numbers in bold are referred to in the text.

### Phylogenetic pattern-mining results

Each of the multi-species gene families was scored for the total number of sequences present from each species and for the presence of inferred duplications in the *Gm*, *Mt*, and *Cf* lineages. Despite substantial completeness of the predicted transcriptome sets from the four species in the study, gene loss and non-recovery were observed in many gene families. In general, this produced uninformative (not scorable) rather than mis-informative results. Patterns with more losses are subject to more uncertainty, since “lossy” patterns may be the degenerate products of different initial patterns. For each of the four hypotheses, we considered patterns with zero through up to three sequence losses, using both automated pattern matching and visual inspection. Count summaries for the automated and manual methods are shown in Table 1. The patterns and counts for all enumerated patterns and trees are shown in Tables S2 and S3.

**Figure 3:**
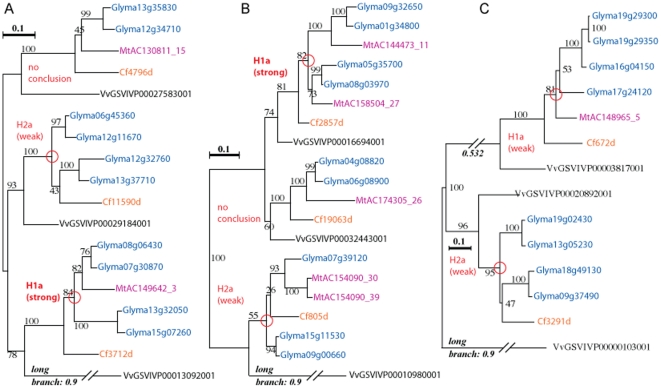
Sample gene phylogenies. Each is consistent with gene triplication before the Vitaceae/rosid I separation, followed by the *Chamaecrista*/Papilionoideae separation, then WGD before the Glycine/Medicago separation; and lastly, WGD in Glycine. At least 18 of the 518 families examined contain similar early triplications with multi-family (Vitis and legume) clades. Inferred duplication timings are shown with red circles. Figure **3A**: Vessicle-associated membrane protein family (cl3975, Table S3). The top clade is ambiguous because of four sequence losses (2*Gm*, 1*Mt*, 1*Cf*), so did not contribute to counts toward any of the four hypotheses. The second is consistent with H2a, but is weak evidence: it has three sequence losses (2*Mt*, *Cf*), and has bootstrap value of 43% on placement of *Cf*. The third clade has two sequence losses (*Mt*, *Cf*), and is consistent with H1a. Figure **3B**: lactoylglutathione lyase protein family. The first clade is “complete” and supports H1a. The second clade is missing four sequences, so contributed to none of the hypotheses. The third clade, with two sequence losses (*Mt*, *Cf*), is consistent with H2a (though is weak evidence, considering the bootstrap value of 26% on placement of *Cf*). Figure **3C**: RmlC-type cupin family (cl5891, Table S3). Neither of the legume clades were counted by the automated methods, but on visual examination, they are weakly consistent with H1a and H2a, respectively. The first clade likely has misordered sequences Glyma16g04150, Glyma17g24120 (which derive from the recent WGD and should group together), and MtAC148965_5. This pattern is closest to H1a if the *Mt* sequence, with bootstrap support of 51%, groups with one of the *Gm* clades. The *Cf* sequence, with high bootstrap value, is in an outgroup position with respect to the papilionoid sequences. The second clade, with two sequence losses (both *Mt*), is consistent with H2a (though is weak evidence, considering the bootstrap value of 47% on placement of *Cf*).

Counts based on automated pattern matching used relatively strict criteria for matches: alternate tree rootings were not considered, and additional gene duplications beyond those in the four models were disallowed. In contrast, visual evaluations of trees allowed for some discretion and interpretation, resulting in higher counts (for example, consideration of additional paralogous duplications, alternate rootings, or multiple informative clades in a tree). Figure 3 shows three trees that illustrate typical clade patterns, including confounding factors such as sequence losses (in all clades except the top in 3B) and probable branch-placement conflicts (e.g. 3C upper), and low bootstrap support for some clades (e.g. 3C lower). In Figure 3C, visual inspection allows scoring of both legume clades whereas automated scoring did not. Nevertheless, these trees together contain six clades scorable with respect to the four models.

Table 1 shows counts of trees or clades consistent with the four models, for both automated and manual counting. For example, considering patterns with losses of up to three sequences from the models, there are 767, 14, 69, and 1 trees consistent with models 1a, 1b, 2a, 2b, respectively (Table 1, numbers in bold). The counting procedures can be seen in Figure 3. The tree in Figure 3A contributes a count of 1 to H1a and also 1 to H2a. No adjustments were made in the counts to reflect “weak” or “strong” cases (Figure 3). However, in general, the H2 counts more often tended to be “weak”, typically due to low-bootstrap placement of one Cf sequence with a ((*Gm*,*Gm*),*Mt*) clade (shown in one clade in each of Figs. 3A–C). In short, there is virtually no support for independent duplication in the *Cf* lineage (model variant b; one of 852 scored clades, with three sequences losses vs. the model). Considering patterns with losses of up to three sequences, there is approximately 11 times more support for “duplication late” (model variant 1 rather than 2).

The patterns of single-species terminal duplications provide an indication of duplications due to polyploidy. Counts are 49, 190, 1749, and 68 for (*Cf,Cf*), (*Mt,Mt*), (*Gm,Gm*), (*Vv,Vv*), respectively (Table 1). The fact that *Gm* shows nine-fold more terminal duplications than the next closest pair, (*Mt*,*Mt*), may be explained primarily by these factors: *Gm* experienced a much more recent second WGD [9]; the genomic sequence used in this comparison is more complete for *Gm* than for *Mt*; and patterns of the old legume duplication that affected *Mt* have been interrupted by subsequent speciation with *Gm*, producing e.g. (((*Gm*,*Gm*),*Mt*),((*Gm*,*Gm*),*Mt*)). The lowest number of terminal duplications, 49, is for (*Cf*,*Cf*). It is worth noting that this number is comparable to 68 for (*Vv,Vv*), and that *Vitis* is known not to have experienced a WGD since the pre-rosid triplication event [26].

In addition to duplication and speciation patterns in the legumes, the multi-species gene trees show intriguing evidence for the pre-rosid triplication [26]. At least 18 trees show clear clade triples, each consisting of a *Vv* sequence and one or more legume sequences. Two such trees are shown in Figure 3. Further, of 335 trees scored with up to two sequence losses, 82 have at least two early-diverging *Vv* sequences indicating at least some widespread duplications prior to the split between *Vitis* and other rosids, and consistent with the pre-rosid triplication.

### Synonymous-site comparisons

**Figure 4:**
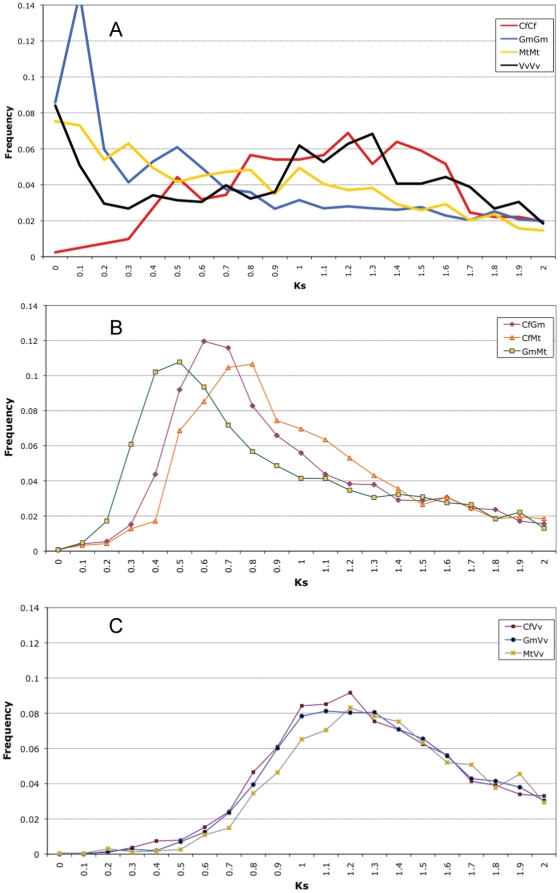
Frequencies of gene pairs by Ks bin from 518 informative multi-species gene trees. Rates of synonymous-site changes (Ks) were calculated for each gene pair from each informative gene. Frequencies were calculated for each Ks value, in intervals of 0.1 Ks, and plotted as a proportion of all such pairs, for the indicated categories. Figure **4A**: self-comparisons (*CfCf*, etc.). Note the recent peak in *GmGm*, corresponding to the ∼10 Mya *Glycine* duplication (value at this peak is 14.6). Figure **4B**: legume-to-legume comparisons (*CfGm*, *CfMt*, etc.). Note the more recent peak in *GmMt* than in either *CfGm* or *CfMt*, as would be expected from the species phylogeny. Figure **4C**: legume-to-Vitis comparisons (*CfVv*, etc.). Note the much “older” peak than in any of the legume self-comparisons, and the possible shift in *MtVv* relative to *CfVv* or *GmVv* – which would be consistent with more rapid change in *Mt* (also evident in Figure 4B).

The 825 scorable trees contained an average of 7.0 sequences per tree – though some trees contained multiple clades that contributed to the tallies. The trees in Figure 3 display typical clade arrangements, although most scored trees contained one or two legume clades rather than the three shown in 3A and 3B. For each tree, we calculated distances based on synonymous and non-synonymous nucleotide changes for each gene pair. Histograms of Ks frequencies from these comparisons are shown in Figures 4A–C. The self-comparisons (*GmGm*, *CfCf*, *MtMt*, *VvVv*) in Figure 4A show two well-defined Ks peaks for *GmGm*, with modes at 0.1, and 0.5, and similar to those reported in Schmutz et al. [9] from the full soybean genome and in earlier studies based on ESTs [30], [31]. These are consistent with WGD in the *Glycine* genus and early in the legumes. The earlier rosid duplication may be swamped out in *GmGm* by duplicates from the more recent peaks; indeed, the early duplication was also not reported by Blanc and Wolfe [31]. A very old, diffuse peak may be evident in both *CfCf* and *VvVv*, with mode around Ks 1.0–1.4.

Recent gene duplications in *MtMt* and *VvVv*, around Ks 0.0–0.1 (Figure 4A), are likely the result of the ongoing recent paralogous duplications that are common in eukaryotic genomes [32]. The much lower proportion of such very recent duplicates in *CfCf* may reflect collapsing of highly similar paralogs during contig construction, or lower rates of recent paralogous duplications, or both. Assuming the lack of a peak in the Ks 0.0–0.1 range is predominantly an artifact of over-contigging, this could conceivably mask a very recent episode of polyploidy in *Cf*. However, karyotype and genome-size considerations argue against recent polyploidy: there are only 8 chromosomes (gametophytic), and the genome size is estimated at only 637 Mb for the related *C. absus* [33]. Further, to collapse duplications from the timeframe of the early episode of polyploidy would require collapsing at the Ks ∼0.5 range. In this Ks range, more than half of the non-coding sites differ; whereas we estimate the rate of nucleotide corrections during contigging to be approximately two bases per thousand, based on the number of changes made by the software exonerate [34] during frame-correction (data not shown). There are also no clear middle-age peaks in the *CfCf* line, suggesting what is evident in the tree patterns: that there has been no WGD in *Cf* since the early rosid triplication. This inference should be tempered, though, by lack of a clear peak in the 0.5–0.7 range for *Mt*. (The early-WGD peak in *Mt* should be shifted rightward (older) with respect to *Gm*, due to the higher rates of nucleotide changes in the *Mt* lineage [5], [35]). The Ks plots for *Mt* and *Cf* – which are aggregate reports of any duplication regardless of its nature or the evolutionary pace of its gene family – should be far less informative about early duplication events than the evaluation of branching orders in individual trees. The absence of a clear early-WGD Ks peak in both *Mt* and *Cf* is not necessarily informative in either direction due to the small number of *Mt* and *Cf* paralogs from these trees – and, therefore, to higher standard error for the Ks estimates. There were, in the 1249 trees from which Ks values were calculated, 891 *Mt* gene pairs, vs 7488 *Gm* gene pairs and 408 *Cf* gene pairs. The small number of paralogs in *Mt* is likely due to a combination of factors: incompleteness of the Mt2.0 genome assembly, and possibly also to greater rates of gene loss or change in that lineage – reported by Cannon et al. [8] and consistent with the higher rate of nucleotide changes in the *Mt* lineage (see below).

In the between-legume comparisons (Figure 4B) the *GmMt* peak, at ∼0.4–0.5, is clearly younger than the *CfGm* or *CfMt* peaks, at ∼0.6–0.7. This corresponds well with the species phylogeny: *Cf* separated before the split of *Gm* and *Mt* [10]. Also evident in Figure 4B is that *CfMt* and *CfGm* peaks differ, at approximately 0.65 and 0.75, respectively. Since these peaks represent the same divergence event (on the basis of duplication and speciation patterns in gene families), *Mt* must be accumulating silent-site changes more rapidly than *Gm*. The finding of more rapid silent-site accumulations in *Mt* has been reported before [5], [6], [31]. This type of rate difference following a common duplication event has been observed in numerous other plant lineages [36], re-emphasizing that despite major differences in age estimates from Ks distributions, the Ks peaks in two species may represent the same event.

In the comparisons of the legume species with *Vv* (Figure 4C), the Ks peaks are diffuse, but all have modes around 1.0–1.4. This is in the same range as the oldest peaks in *CfCf* and *VvVv* (Figure 4A), suggesting again that the oldest WGD in *Cf* occurred in the same time frame as the separation of Vitaceae from other rosids. It is worth noting that the *MtVv* curve is shifted (by ∼ +0.1) with respect to the *CfVv* and *GmVv* curves, again consistent with more rapid accumulation of silent-site mutations in *Mt* following the *Cf/Mt* and *Cf/Gm* speciations.

## Discussion

The *Chamaecrista* genome does not show evidence of a whole genome duplication other than one that occurred in a distant pre-rosid ancestor. In particular, our results do not support that the *Chamaecrista* lineage experienced the polyploidy event found in papilionoid legumes such as *Medicago* and *Glycine*. This finding adds considerably to our understanding of the legumes, which comprise the third largest family of flowering plants and are of great economic and ecological importance [37], [38], [39].

The legumes are intriguing for their capacity for symbiotic nitrogen fixation, for their great diversity, and for their rapid radiation and global spread following the origin of the family, approximately 60 Mya [10]. However, nodulation and diversity are unevenly distributed across the family. Traditionally, the family has been classified into three subfamilies of very unequal sizes [phylogenetic numbers and summary from 40]: Papilionoideae (the typical “beans” such as pea, lupine, soybean, and peanut, with 478 genera and an estimated 13,805 species); Mimosoideae (e.g., acacia; 92 genera and 3,271 species); and Caesalpinioideae (171 genera and 2,251 species). Mimosoideae and Papilionoideae are monophyletic groups, whereas Caesalpinioideae is polyphyletic. The largest group of caesalpinioid legumes (59 genera and 1,094 species) are part of the “mimosoid” clade that also includes Mimosoideae, and is sister to Papilionoideae. Thus, the two largest radiations of the family—85% of the genera and 94% of the species—belong to these two sister clades, which diverged from the remaining legume lineages shortly after the origin of the family [10], [39].

Nodulation is confined to these two largest clades, but is not universal in either [41]. Some early diverging lineages of Papilionoideae do not nodulate, suggesting a possible origin of nodulation after origination of the subfamily. Although most surveyed species in Mimosoideae nodulate, and nodulation occurs in the closest caesalpinioid relatives of Mimosoideae, nodulation is confined to only a few genera outside of this group, one of them being *Chamaecrista*. Given this distribution, it is therefore possible that nodulation has originated multiple times in the family – though multiple-loss scenarios are also plausible [21].

Thus, in legumes we have nodulation and a polyploidy event that could be correlated with it, and either of these phenomena could be correlated with extensive speciation. Is there a causal relationship? Our data are not consistent with a single polyploidy event in the ancestor of all legumes, and thus refinement of these hypotheses is required. Because *Chamaecrista* does not share the papilionoid polyploidy event found in *Medicago* and *Glycine*, that genome duplication must have occurred either in the common ancestor of all papilionoids or early in the radiation of that subfamily. Details of the early phylogenetic history of the papilionoid clade remain unclear due to its apparently rapid radiation [10], [42]. The fact that the polyploidy event is shared by *Glycine* (millettioid clade) and *Medicago* (Hologalegina) means that it took place before 54 Mya, during the initial rapid radiation of the papilionoids that began around 59 Mya. Other major clades that comprise that radiation are now known to have undergone the same polyploidy event found in the genomes of *Glycine* and *Medicago*. These include the dalbergioid *Arachis* [12] and possibly the genistoid *Lupinus* [13]. A small number of additional early-diverging papilionoids [10], [22] also remain to be sampled for this event, including the *Swartzia* and *Cladastris* clades (Figure 1). Thus, regardless of whether every papilionoid taxon experienced the polyploidy event, there is a strong correlation between polyploidy and evolutionary radiation—the legume lineage with the largest number of genera and species is polyploid. It is also among the “basal papilionoid” group of genera that the non-nodulating members of the subfamily occur, e.g. the vataireoids and the *Cladastris* clade [22], [42], [43], [44], [45]. Therefore, it is possible that polyploidy and nodulation in the papilionoids are correlated, and thus there could be a causal relationship (though not a necessary one). Determination of early papilionoid phylogeny, more information on the distribution of nodulation in many mostly tropical woody genera, and detailed studies of individual gene families involved in nodulation could provide the data needed to test this hypothesis.

The absence of polyploidy in *Chamaecrista* is what eliminates any *necessary* correspondence between nodulation and polyploidy in legumes. There has been neither a single polyploidy event in the common ancestor of all nodulating species (i.e., in the common ancestor of the papilionoids and the mimosoid clade), nor two independent polyploidy events in papilionoids and *Chamaecrista* (or the common ancestor of *Chamaecrista* and the Mimosoideae and its nodulating close relatives). Whether the common ancestor of the Mimosoideae and their close relatives (excluding *Chamaecrista*) underwent a whole genome duplication awaits genomic studies in these taxa; the base chromosome number of *x*  = 13 or 14 suggests that the mimosoids may be polyploid. If so, the mimosoid crown polyploid event occurred sometime during the past ca. 55 Mya, since its common ancestor with *Chamaecrista*. In any case, the lack of polyploidy for *Chamaecrista* means that, in the sense of a family-defining event, the legumes are not fundamentally polyploid as a group.

It remains to be seen whether nodulation is homologous in *Chamaecrista* and other legumes, such as other members of the mimosoid clade or in the more distantly related papilionoids. Nodulation has arisen multiple times in members of the rosid I “nitrogen-fixing clade” including the legumes, suggesting a predisposition for nodulation that evolved in the non-nodulating common ancestor of this large group [18]. This same predisposition could underlie multiple origins within the legumes. However, neither in the case of the nitrogen-fixing clade nor in the legumes as a whole has polyploidy been directly associated with the origin of nodulation.

If genome duplication was not required for origination of nodulation in the legumes, is there anything about *Chamaecrista* nodules that might clarify our understanding of nodule evolution? Indeed, *Chamaecrista* nodules appear to exhibit a number of putatively ancestral nodule characters relative to nodules of most papilionoid lineages. Rhizobia appear to enter via epidermal cracks [41], [46]. Shortly after entry, the bacteria are surrounded by a plant membrane; this modified infection-thread-like structure, called the “fixation thread” by Sprent [41], varies substantially among the evaluated *Chamaecrista* species. Naisbitt et al. [46] describe thick-walled, persistent fixation threads (the tree species *C. ensiformis*); thin-walled threads (the perennial shrub *C. desvauxii*); and transient threads that release bacteria into the cytoplasm (*C. fasciculata* and 10 other species sampled, including perennial and annual shrubs and herbs). In most papilionoid genera, nitrogen fixation occurs only after bacteria are released from the infection thread. In contrast, nitrogen fixation occurs within the persistent fixation thread in those *Chamaecrista* species that contain this structure. The crack entry seen in *Chamaecrista* (i.e. infection not via root-hair), followed by variable response by the plant (formation of a persistent vs. a transient membrane), both suggest a less elaborate process than the process found in most papilionoids, involving root-hair curling and formation of an infection thread in advance of the bacteria's path [44], [47]. Even in legumes that normally produce nodules through infection threads, rhizobial infection and subsequent nodulation via crack entry has been observed; examples include both papilionoids (e.g., *Trifolium, Lotus*) and mimosoids (e.g., *Neptunia*, *Mimosa*) [reviewed in 41], [ 48], [ 49]. This suggests that the ability to nodulate via crack or apoplastic entry may be a more ancestral state to which other legumes may default under some conditions [41].

Thus, a revised hypothesis in light of the *Chamaecrista* information is that nodulation or proto-nodulation existed before the papilionoid WGD, perhaps utilizing crack- or epidermal entry, with a plant response involving membrane production around the invading rhizobia. This remained the evolutionary path in most of the caesalpinoid, the mimosoid and in the dalbergioid and genistoid legumes (the latter being papilionoids, but perhaps retaining more ancestral nodule developmental patterns). Following WGD in the lineage leading to the papilionoids or early in that lineage, duplicated genes facilitated elaboration of biochemical responses and developmental structures (e.g. infection thread and root-hair involvement). Thus, although WGD was evidently not required for the evolution of nitrogen-fixing nodulation, it may have fostered development of several elaborate papilionoid nodule forms, and may also have helped spur the rapid radiation and ecological success of the papilionoids. Testing of these hypotheses will require further examination of gene function in the context of gene duplication patterns in species exhibiting various nodule characteristics across the legumes.

## Materials and Methods

### Tissue collection

Plants from a *C. fasciculata* ecotype collected in Minnesota (MN98, originally JEM98 obtained from J. Etterson) were grown for tissue in a controlled environment growth room. Tissues were collected from 14 conditions, including root, shoot, nodule, and inflorescence tissues at several time points (Table S1). Tissue was prepared for mRNA library production. For shoot tip tissue, seeds were surface sterilized, scarified with sandpaper, and germinated on moistened filter paper in Petri dishes in a growth chamber with the following conditions: 16 h high intensity light (21°C), 8 h darkness (21°C). Germinated seedlings were placed in pots containing a soilless media (Metromix 360, Fafard) and grown in a growth room with the following conditions: 8 h high intensity light (21°C) followed by 10 h day extension lighting with incandescent light (18.5°C), 6 h darkness (18.5°C). Shoot tips (apical tips above the last expanded leaf) were harvested from age-staged plants. For nodules and root tissue, germinated seeds were placed on a sterilized mixture of vermiculite:perlite (1∶1) moistened with 1/4 strength Hoagland nitrogen-free media, pH 7.0 (Plant Media) and inoculated at planting with *Rhizobium* Cowpea Type (Nitragin EL, EMD Crop Biosciences, Inc., Milwaukee, WI) and grown in a growth chamber with the following conditions: 16 h high intensity light (21°C), 8 h darkness (21°C). Plants were watered with 1/4 strength Hoagland nitrogen-free media, pH 7.0, and monitored for root and nodule formation.

RNA was isolated from developmentally staged *C. fasciculata* (MN98) shoot tips, roots, root tips, nodules, and nodules with associated root tissue at the point of attachment (Table S1, showing conditions and tissue photographs). RNA for the mRNA library production was extracted from tissue with an RNA Progreen RNA Isolation Kit (Q-Biogene). The RNA was then treated with DNAse (Turbo DNA-free, Ambion) and stored at −8°C prior to shipment to NCGR for mRNA library production.

### Sequencing

Libraries were sequenced using two methods: with Roche 454 Titanium on pooled RNA from all 14 tissues to generate long reads to serve as alignment templates; and with the Illumina Genome Analyzer to generate high sequence coverage and transcript counts (for use in analyses to be published separately).

For the Roche 454 Titanium sequencing, cDNA was synthesized with the following adaptors, and subsequently sheared during 454 library prep.

5′end-AAGCAGTGGTATCAACGCAGAGTGGCCATTACGGCCGGG-cDNA-AAAAAAAAAAGAAAAAAAAACAAAACATGTCGGCCGCCTCGGTCTCTA-3′end

The total number of reads in the bulk run was 950,227 with an average read-length of 344.69 bp and a total number of bases of 327,532,623.

For short-read generation (Illumina sequence), whole transcriptome shotgun (WTS) libraries were generated, and were sequenced on an Illumina Genome Analyzer to a read length of 46 base pairs. The average number of reads per library was 9.428 million, and total reads were 132 million, and 6 billion DNA bases (Table 1).

### Sequence assembly and processing

Assembly proceeded by contigging the 454 reads, followed by several rounds of contig extensions. The 454 reads were contigged using a custom perl pipeline and Blat [50] to bin homologous sequences, then cap3 [51] to align sequences in contigs. Illumina reads were filtered for quality as follows. Low-scoring right tails were trimmed from any bases with phred score <4 (after conversion to phred scores with a custom perl script). Additionally, the following low-scoring sequences were removed: sequences consisting of greater than 66% of poly-A -T -G or -C; and sequences with fewer than 30 high quality bases.

Contig generation and extension with Illumina sequence proceeded using VCAKE [52], adding sequences in four batches (to avoid computer memory limitations in the 32 bit perl implementation of VCAKE). Each round used VCAKE parameters -k 42 (i.e. use 42 nucleotides from each sequence); -n 21 (percent overlap required; default is 18). The first VCAKE round extended from the 454 contigs, and next two round extended from the preceding contigs. After the third round, contigs were collapsed using cap3 [51], using default parameters. In the final round of Illumina extensions, contigs and singletons from cap3 were extended with the remaining batch of Illumina reads, then re-collapsed with a final round of cap3. This set of preliminary contigs consisted of 54,903 contigs, with average length 599 nt.

Evaluation of the initial contigs by homology comparisons of the sequences with themselves indicated under-contigging, with highly similar sequences probably remaining from allelic variants (from heterozygous source DNA), splice variants, and minor sequencing variants. Therefore, preparatory to alignments and tree construction, we used two additional rounds of contigging with cap3, followed by conceptual translation using exonerate [34] to align each *C. fasciculata* sequence in-frame to the most similar soybean peptide sequence from the Glyma1.01 annotation. We used the exonerate alignments and custom perl scripts to remove single-base insertions responsible for probable frame shifts, and a final run of cap3 to re-collapse in-frame sequences resulting from the exonerate comparisons. The resulting file has 21,781 sequences, with average length 463 nt. Of these sequences, 90.7% are without internal stop codons in frame 0. The remainder may either be ORFs in other frames, or may contain frame shifts. Contig sequences are available in Supplementary File S1.

### Clustering, alignments, and phylogeny construction

Sources of predicted gene sequences for the comparison genomes were as follows: for *Mt*, the *Mt*2.0 mRNA sequences from http://www.medicago.org/genome/downloads/Mt2/ [29]; for *Vv*, the *Vitis*_vinifera_mRNA_v1.f file from http://www.genoscope.cns.fr/externe/Download/Projets/Projet_ML/data/annotation/ [26]; and from *Gm*, the Glyma1.cDNA.fa file from the Glyma1.01 genome build and annotation from http://www.phytozome.org/soybean [9].

Gene families were generated by first making all-by-all homology comparisons among cDNA sequences in the four genomes, then constructing single-linkage clusters. Homology searches were conducted with NCBI blastall [53], with parameters -p blastp -e 1e-10 -b 10. Blast hits were further filtered to require > = 50% identity and match length > = 60 aa (180 nt). Clusters were also filtered to identify likely informative families prior to alignment and tree-making. Filtering for retaining clusters used the following count restrictions *Cf* > = 1, *Gm* > = 2, *Mt* > = 1, *Vv* > = 1, *Cf*+*Gm*+*Mt*+*Vv* < = 100.

Maximum likelihood phylogenies were calculated on in-frame codon alignments with RAxML [54], using the GTR model with four gamma categories for rate heterogeneity (with parameters estimated from the alignment by RAxML). Trimmed alignments and trees are available in Supplementary File S2.

### Synonymous site analysis

Counts of changes per synonymous site were calculated between all sequence pairs in each gene family, based on in-frame codon alignments, implemented in the SNAP program [55]. The Ks values in SNAP are calculated using the method of Nei and Gojobori [56], with Jukes-Cantor correction for multiple hits in the proportion of synonymous substitutions.

### Quantification of tree patterns

Trees were evaluated for patterns consistent with hypotheses 1 and 2, and variants a and b (Figure 2), using two methods: first, pattern searches, implemented in Perl regular expressions, to count patterns consistent with the hypotheses (allowing for prescribed numbers of gene losses); and second, manual examination and scoring of all trees. For the automated approach, trees were first rooted on an arbitrarily chosen *Vv* sequence. Branch lengths were stripped, sequence names were simplified, and clades were rotated to reduce the number of potential patterns. All patterns used for these counts, as well as the perl counting program, are provided in Table S2. For the manual approach, trees were rooted by inspection, with the goal of selecting a plausible root by reconciling the gene tree with the known species tree. In practice, the selected root was usually either a *Vv* clade or a midpoint between two or more multi-species (*Vv* + legume) clades. For hypotheses 1 and 2, all possible deletion variants up to loss of two *Gm* and one *Mt* sequence were considered, as shown in Table 1. Also, for hypothesis 2 (duplication before the *Cf*,(*MtGm*) separation), loss of one *Cf* gene was considered.

## Supporting Information

Table S1 Summary of C. fasciculata tissues used for RNA isolation. MN98 refers to the Minnesota ecotype that was sequenced. Age refers to the number of expanded leaves present on plants from which shoot tips were isolated.
(3.21 MB DOC)

Table S2 Automated counts of patterns found in 1,249 informative phylogenetic trees. The indicated patterns are simplified text representations of phylogenetic tree topologies. The enumerated counts include all variants consistent with models H1a, H1b, H2a, H2b, allowing up to four missing sequence from the full “canonical” patterns. For example, ((((G,G),M),((G,G),M)),C) is the representation of model H1a, assuming outgroup rooting by either Vv or other multi-species clades. Clades in all trees were first modified where necessary to ensure that variants such as ((G,G),M) and (M,(G,G)) were both counted.
(0.05 MB XLS)

Table S3 Manual counts of patterns found in 1,249 informative phylogenetic trees. Columns D-G show the numbers of sequences missing relative to the canonical pattern for the indicated model (H1a … H2b).
(0.25 MB XLS)

File S1 Chamaecrista fasciculata contig sequences.
(2.09 MB GZ)

File S2 Alignments and trees. Trimmed alignments and trees for all 1249 trees described in the paper.
(2.55 MB GZ)
