# Supplementary material for: Polyploidy Did Not Predate the Evolution of Nodulation in All Legumes
Source: PLoS One. 2010 Jul 16;5(7):e11630. doi: 10.1371/journal.pone.0011630 (PMC2905438; doi:10.1371/journal.pone.0011630)
Supplement: Table S1 — Summary of C. fasciculata tissues used for RNA isolation. MN98 refers to the Minnesota ecotype that was sequenced. Age refers to the number of expanded leaves present on plants from which shoot tips were isolated. (3.21 MB DOC) [file pone.0011630.s001.doc]

**Table I. Summary of *Chamaecrista* Tissues used for RNA Isolation. MN98 refers to the Minnesota ecotype that was sequenced. Age refers to the number of expanded leaves present on plants from which shoot tips were isolated.**

| **Library Code** | **Tissue/Library** | **Fresh Weight (g)** | **No. of Plants Used in the Isolation** | **Photographs of Plant Tissue** |
| --- | --- | --- | --- | --- |
| 1Sa | Shoot tips from 4 day imbibed seeds of the MN98 ecotype | N/A | 25 | 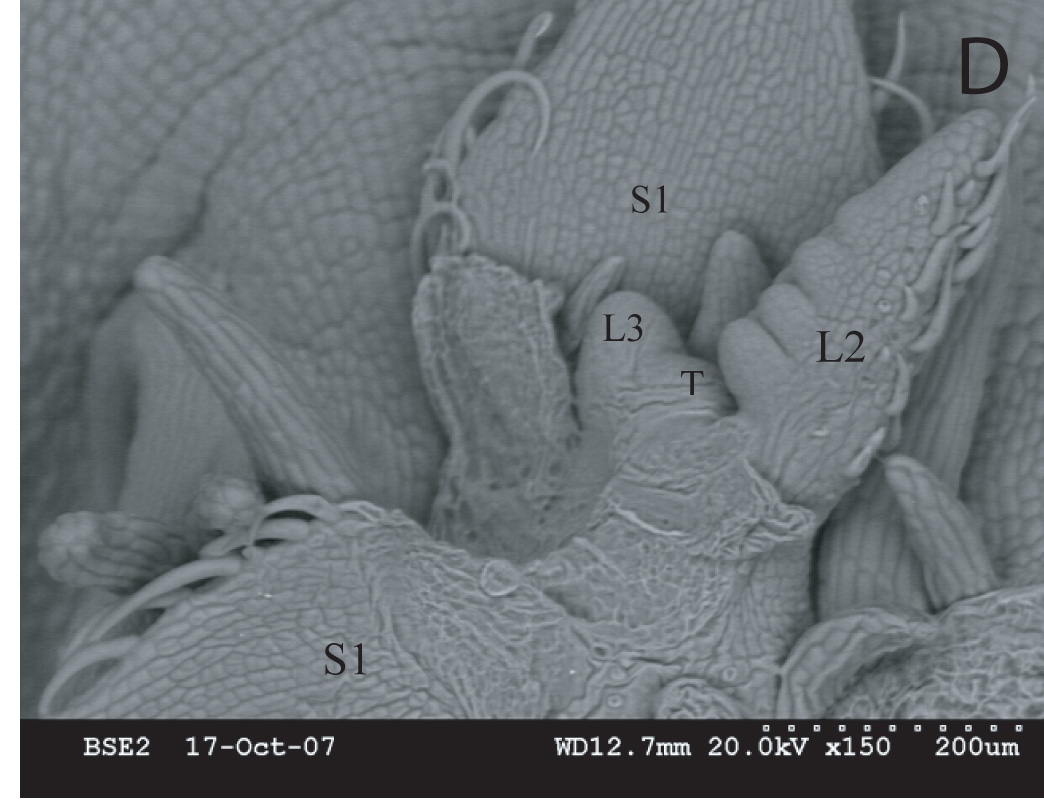 |
| 1Sb | Age 2 Shoot Tips from the MN 98 Ecotype | 0.07 | 27 | 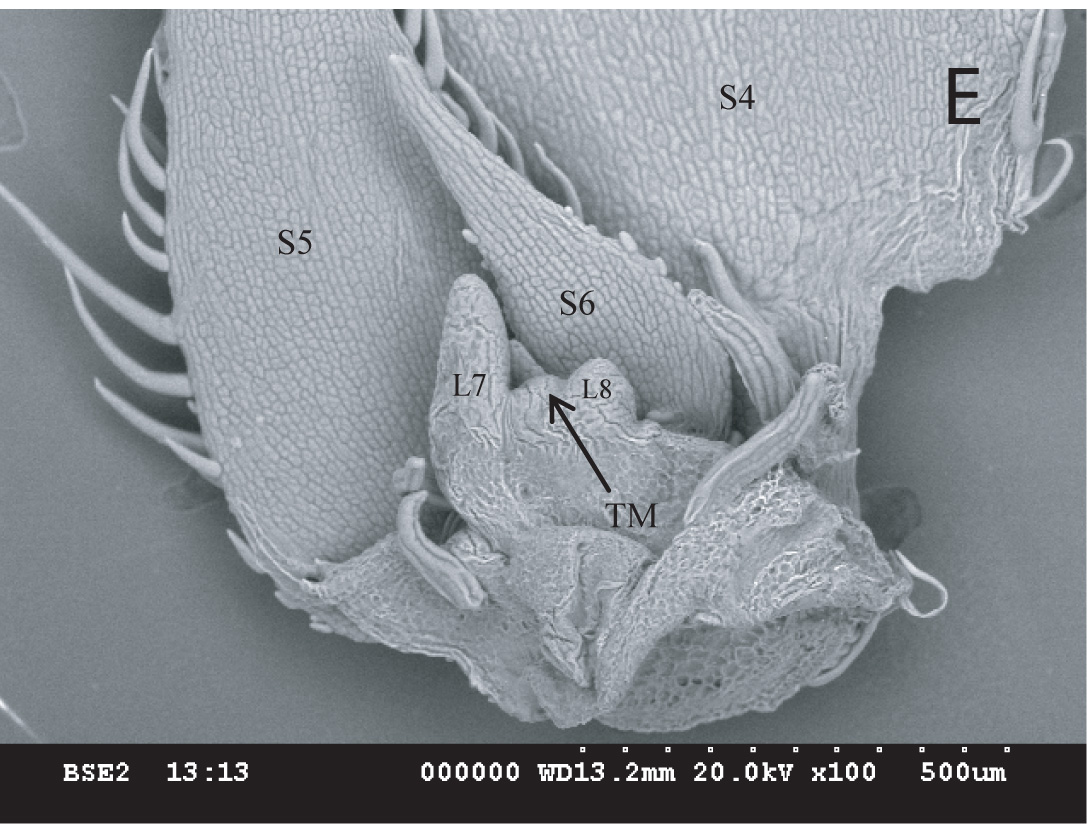 |
| 1Sc | Age 4 Shoot Tips from the MN98 Ecotype | 0.09 | 10 | 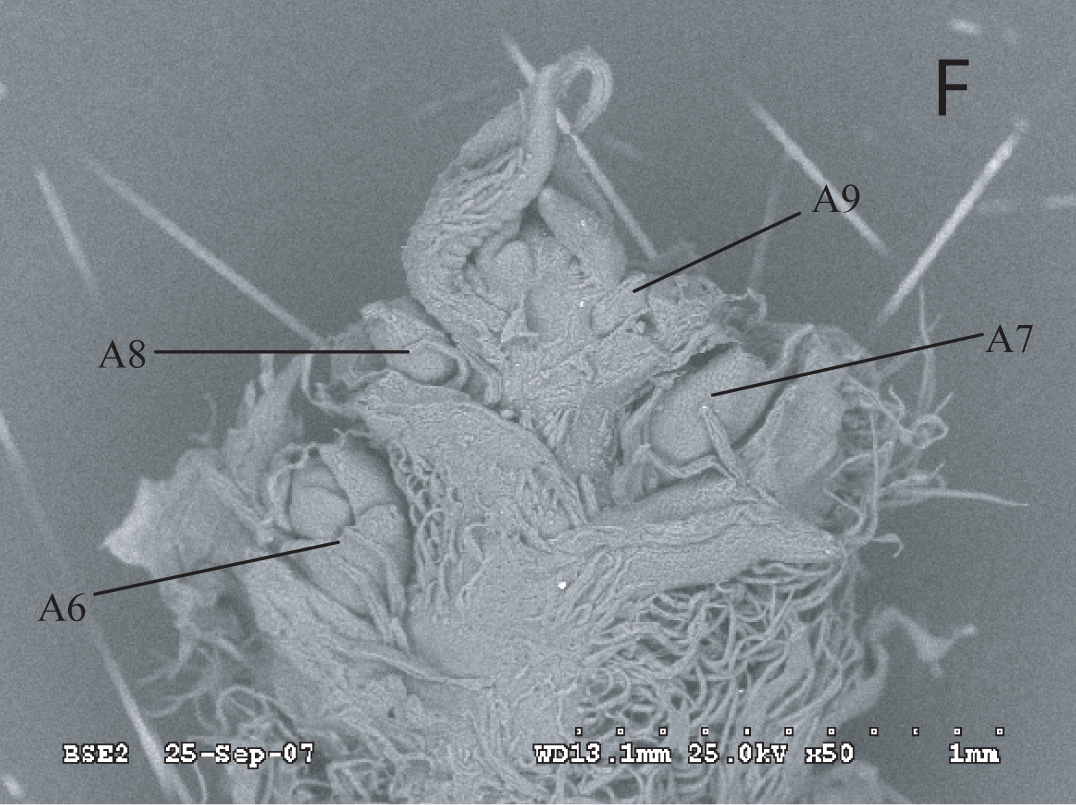 |
| 1Sd | Age 6 Shoot Tips from the MN98 Ecotype | 0.12 | 9 | 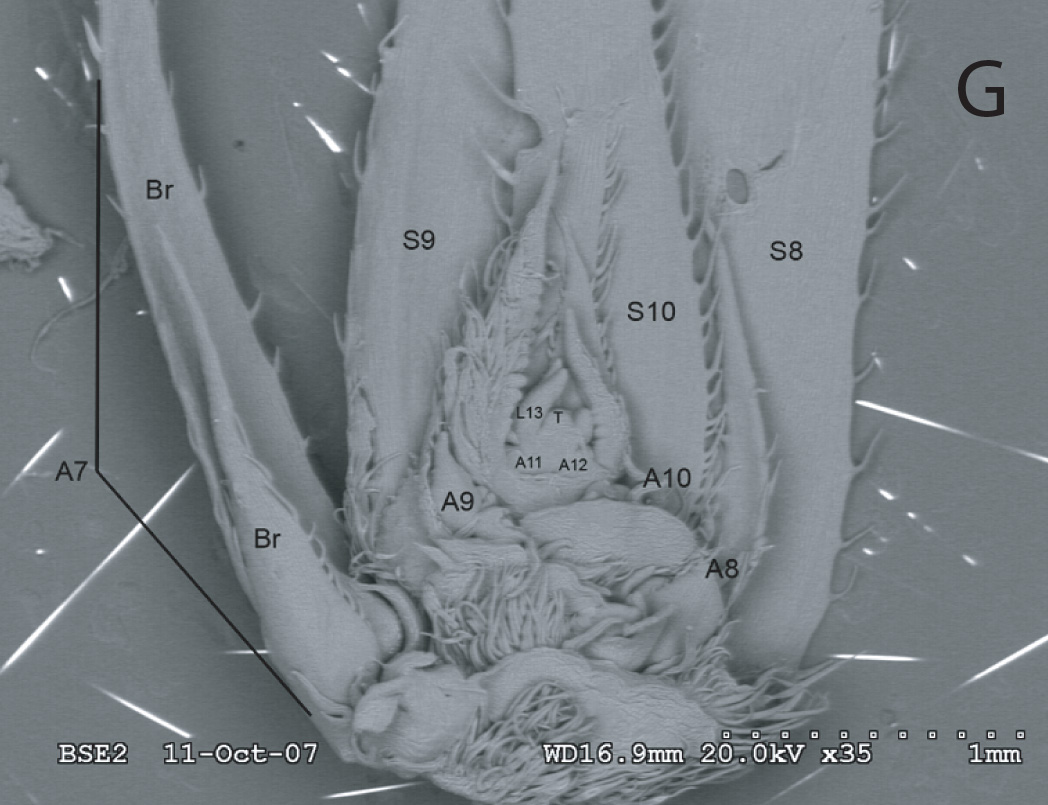 |
| 1Se | Age 8 Shoot Tips from the MN98 Ecotype | 0.13 | 5 | 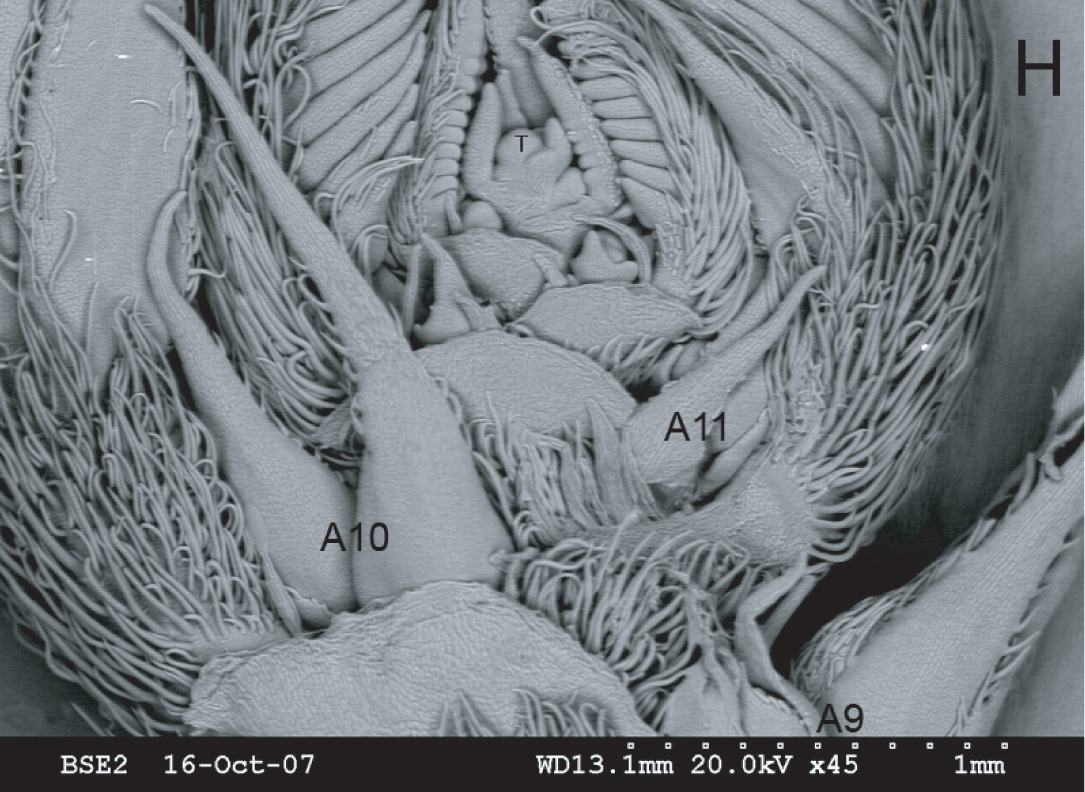 |
| 1Sf | Shoot tips from plants ages 12-16 from the MN98 ecotype | 0.05 | 7 | 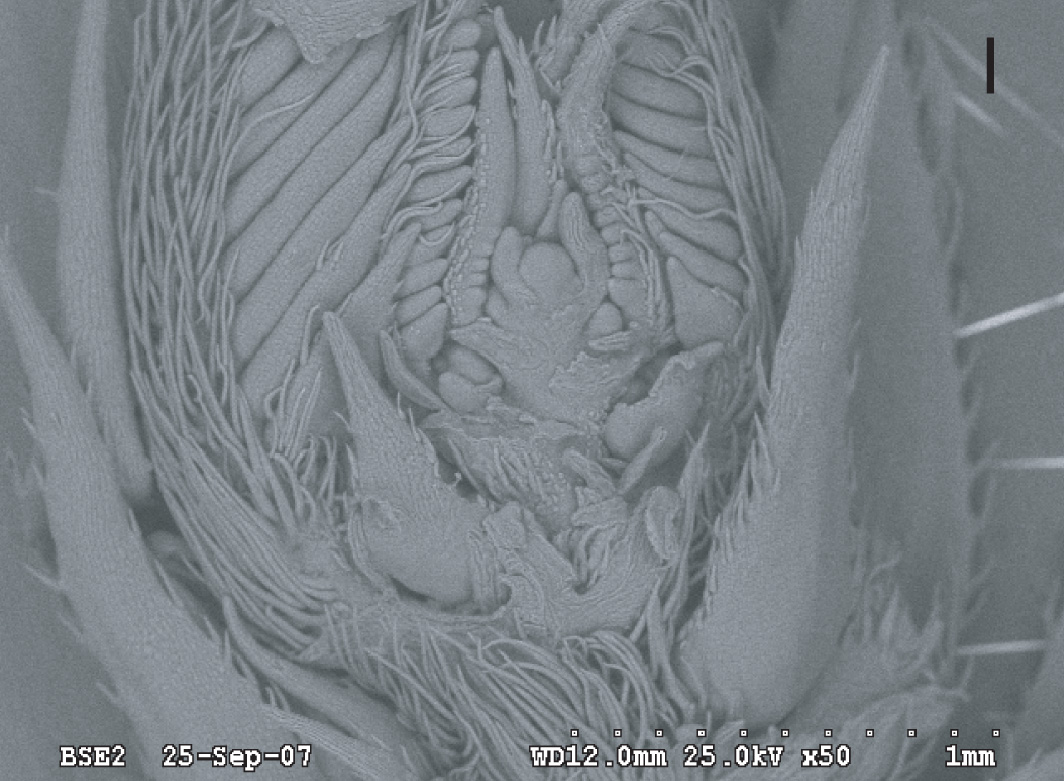 |
| 1Sg | Shoot tips from older reproductive shoot apices (ages 18-21) of the MN98 ecotype | 0.09 | 4 | 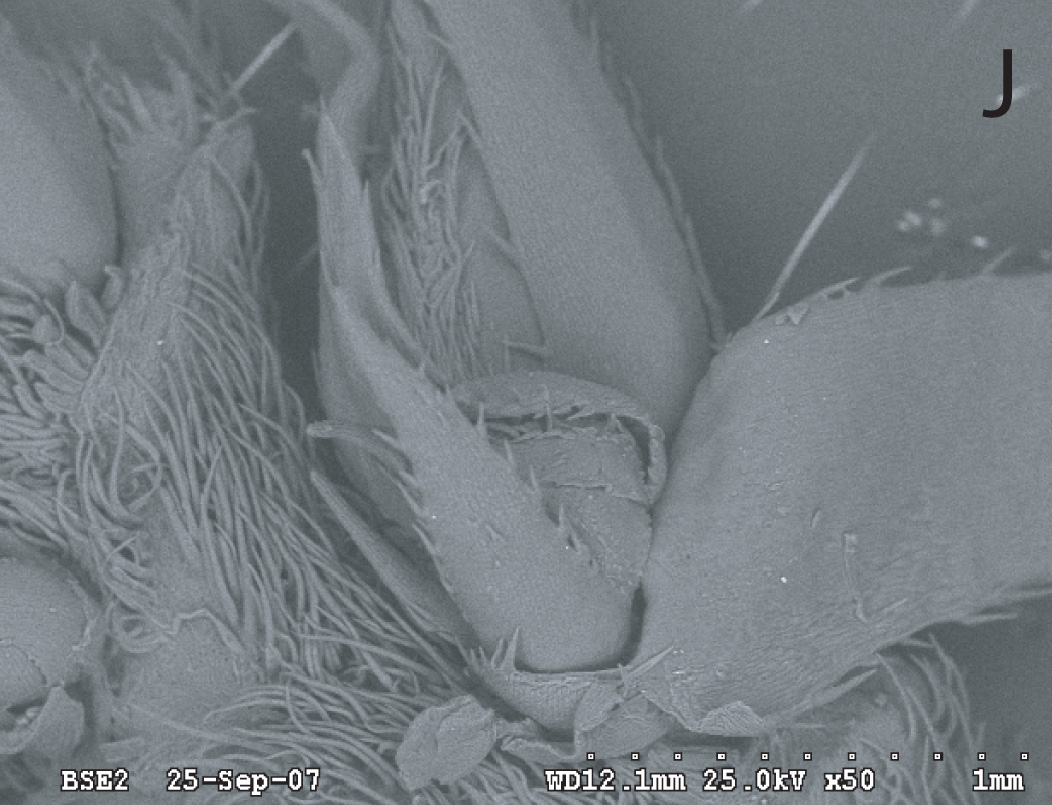 |
| Nm | Whole nodules (14) from the MN98 ecotype | 0.08 | 7 | 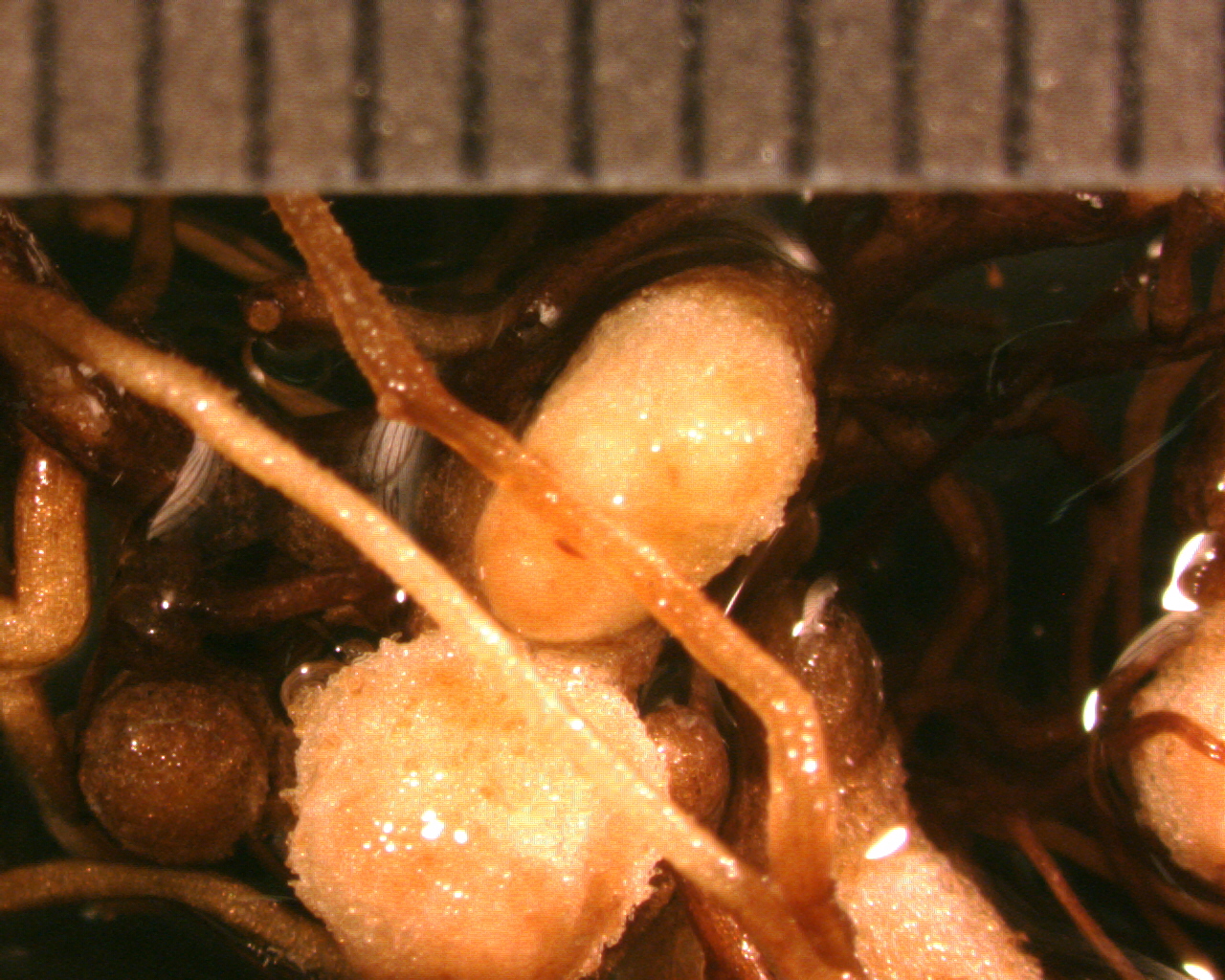 |
| N+Rn | Nodules plus roots of the MN98 ecotype | 0.09 | 5 |  |
| No | Non-scenescent portions of nodules from the MN98 ecotype | Dissected from 0.08 g of nodules | 7 | 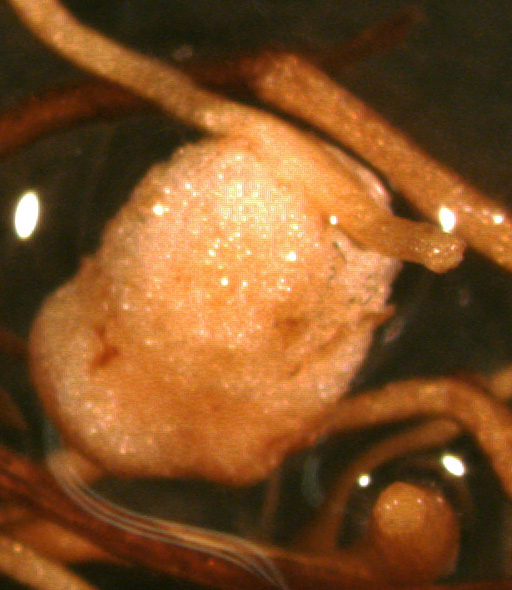 |
| Np | Senescent portion of the nodules from the MN98 ecotype | Dissected from 0.08 g of nodules | 7 |  |
| 1Rj | Root tips (20) of the MN98 ecotype | N/A | 5 | 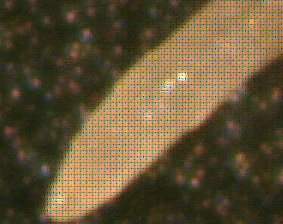 |
| 1Rk | Non-branched roots from the MN98 ecotype | 0.23 g | 20 |  |
| 1Rl | Branched roots from the MN98 ecotype | 0.32 g | 11 |  |
